# The Governor and the Asylums

**Published:** 1900-02

**Authors:** 


**LAW-MAKING A LA TEXAS.**

THE GOVERNOR AND THE ASYLUMS. It will be remembered that the Legislature last winter, in regular session, cut off the subsistence (at State expense) of the Superintendents and their families, a consideration as part payment for their services, to eke out the miserable little salary allowed them, and a custom which had obtained from the founding of those institutions. This was done upon the Governor's express request, in his message. He approved the bill when it had been passed, and it became the law. It seems he has awakened to a realization of the fact that men competent to manage such institutions cannot be picked up every day, and are not willing to work for $2000 a year and find themselves. It is unfortunate that his foresight was not as good as his hind sight appears to be. Superintendent Wilson of the North Texas Asylum, who gave up a business at Sherman worth treble the salary, accepted the appointment at the Governor's urgent solicitation. He promptly resigned when the bill referred to was signed, and although he has insisted upon the acceptance of his resignation he is still in charge—at a heavy pecuniary loss to himself, and so far, no one has been found who will accept the place. Other superintendents did not resign, for reasons best known to themselves; but at the expiration of their time I dare say there will be no rush for their places; they will go begging. The Governor apparently realizes this fact, and having called the Legislature to meet in special session (at taxpayers expense) he includes in his message a recommendation to raise the salaries of the superintendents. He says:

"A consideration of the readjustment of the salaries of the superintendents of the three lunatic asylums is earnestly recommended. Their compensation, as now fixed, is but $2000 per annum. Heretofore, the custom has been for them to use without charge, for the support of themselves and their families, supplies purchased for the maintenance of the institutions. This custom has been abrogated by law [at his request.—ED.].

"When it is considered that these officials should be gentlemen of not only the highest personal and professional character, but of excellent administrative ability also, it can not but be admitted that their salary, as at present constituted, is altogether inadequate to the kind and amount of the service to be rendered and of the responsibility imposed."

The Legislature was called to meet in extra session ostensibly to overhaul and revolutionize the tax laws. With that we have nothing to do; but here is what the *Austin Daily Statesman* says:

"Governor Sayers sent a message to the Legislature on the opening day, in which he stated that the State must have more taxes, and suggested the tax commission bill as a means of securing the same. The next day he sent a second message in, saying that the State had so much money in its treasury that he wanted the taxes reduced."

In the Governor's message he points out that the appropriations made by last Legislature are not near enough to run several departments; that for the several asylums being largely inadequate. It is a most remarkable policy, in the interest of "economy," it is alleged, to cut down salaries and appropriations generally, for the maintenance of our charity institutions, and other necessary expenses, and in a few months find that it is insufficient, and then to assemble the Legislature to undo it, or to do what they should have done at the regular session.

